# Energy-Constrained Design of Joint NOMA-Diversity Schemes with Imperfect Interference Cancellation

**DOI:** 10.3390/s21124194

**Published:** 2021-06-18

**Authors:** Fulvio Babich, Giulia Buttazzoni, Francesca Vatta, Massimiliano Comisso

**Affiliations:** Department of Engineering and Architecture, University of Trieste, Via A. Valerio 10, 34127 Trieste, Italy; gbuttazzoni@units.it (G.B.); vatta@units.it (F.V.); mcomisso@units.it (M.C.)

**Keywords:** random access, slotted Aloha, NOMA, packet diversity, energy constraints

## Abstract

This study proposes a set of novel random access protocols combining Packet Repetition (PR) schemes, such as Contention Resolution Diversity Slotted Aloha (CRDSA) and Irregular Repetition SA (IRSA), with Non Orthogonal Multiple Access (NOMA). Differently from previous NOMA/CRDSA and NOMA/IRSA proposals, this work analytically derives the energy levels considering two realistic elements: the residual interference due to imperfect Interference Cancellation (IC), and the presence of requirements on the power spent for the transmission. More precisely, the energy-limited scenario is based on the relationship between the average available energy and the selected code modulation pair, thus being of specific interest for the implementation of the Internet of Things (IoT) technology in forthcoming fifth-generation (5G) systems. Moreover, a theoretical model based on the density evolution method is developed and numerically validated by extensive simulations to evaluate the limiting throughput and to explore the actual performance of different NOMA/PR schemes in energy-constrained scenarios.

## 1. Introduction

The design of an effective technique for handling a large amount of uncoordinated traffic is still an open issue both for Fifth and Sixth Generation (5G/6G) communication systems, whose final aim is the provisioning of a pervasive space/terrestrial scenario implementing the Internet of Things (IoT) paradigm [1,2,3,4]. Existing networks manage the distributed load by adopting the well known Slotted Aloha (SA) protocol [5], for which several extensions have been proposed [6,7,8,9,10,11,12,13]. The SA performance may be in fact significantly improved by introducing energy diversity and Interference Cancellation (IC), whose combination leads to the Non-Orthogonal Multiple Access (NOMA) approach [6,7,8]. In a NOMA scheme, each user randomly selects an energy level by knowing the allowed ones, but not knowing those selected by the other users. In particular, the levels are chosen so that, if at most *L* users send their packets in a given slot using different levels, all the packets can be decoded.

Beside NOMA, another approach exploits IC, but relying on Packet Repetition (PR), that is, considering the transmission of a certain number *M* of replicas of a generated packet [9,10,11,12,13]. The *M* value may be fixed, as in the Contention Resolution Diversity SA (CRDSA) scheme [9], where M=2, or may be a Random Variable (RV), as in the Irregular Repetition SA (IRSA) protocol [10]. The basic mechanism allowing packet diversity to properly operate relies on the possibility, still enabled by IC, that the packets transmitted in an uncollided slot may be successfully detected and then used to remove their replicas present in other slots. Even if the repetition-based solutions have been further extended to include priority [11], load control [12], and coding [13], the recent introduction of the IRSA algorithm in the DVB-RCS2 standard [14] has made this latter strategy a more immediate candidate for managing the random access procedure in 5G/6G networks.

Rather than being alternative, the NOMA and PR approaches are instead complementary. Accordingly, some proposals have explored the joint usage of energy and packet diversity [15], while specific investigations for the NOMA/CRDSA and NOMA/IRSA schemes have been considered [16,17,18]. These latter studies have proved the reciprocal benefits among NOMA and PR, but their joint applicability in a real network requires a further deepening of some practical aspects. The first one concerns the reception criterion, which usually relies on the simplified erasure model and not on the more realistic capture one [19,20,21]. The second aspect involves the assumption of perfect IC, which does not occur in an actual receiver [22,23,24,25,26], and leads to two main consequences, related to the need of increasing the energy separation to allow the decoding of packets using different levels. Firstly, the imperfect IC may reduce the achievable performance, which also depends on the available energy. In a PR scheme, the energy requirements are more severe, the number of packets that have been detected in other slots being unpredictable, but the residual energy of which may prevent the correct detection of packets in the imperfectly cleaned slots. As a second consequence, imperfect IC may prevent the complete successful delivery of the packets, which may be instead assumed in a PR scheme when the total load lies below a given threshold [9,10]. A further aspect that is often neglected in the analysis of NOMA-based protocols concerns the control of the power consumption [27,28,29], representing a relevant issue for 5G/6G networks, which are expected to be developed in agreement with the green communications paradigm.

In agreement with the above discussion, this paper presents the design of energy-constrained NOMA/CRDSA and NOMA/IRSA schemes able to sustain a desired level of imperfect IC. To this aim, a parameterized model is derived to evaluate the energy levels according to the PR scheme, the amount of residual interference, and the available average energy. To check the benefits of the conceived solution, a theoretical method, validated by extensive simulations, is developed to estimate the throughput and determine the conditions at which the limiting performance, obtained with perfect IC, is approached with imperfect IC. Besides, the proposed design is also tested in the presence of a Quadrature Phase-Shift Keying (QPSK) modulation and adopting a discretized set of channel code rates to consider the realistic situation in which a limited set of transmission modes is available. In particular, the key contributions of the presented study are:

- The development and evaluation of an energy design method for joint NOMA/PR schemes able to sustain a desired level of imperfect IC;
- The derivation of a parameterized model to assess the energy thresholds at which the capability of sustaining an imperfect IC improves significantly;
- The testing of the proposed design in the presence of a QPSK modulation by adopting a discretized set of channel code rates with the aim of verifying its usefulness in realistic scenarios.

The paper is organized as follows. Section 2 introduces the system model. Section 3 presents the energy level design. Section 4 describes the throughput analysis. Section 5 discusses the numerical results. Finally, Section 6 summarizes the most relevant conclusions.

## 2. System Model

Consider a distributed wireless network in which *H* contending users send packets to a common destination. The packet arrival is described by a Poisson process and the time domain is subdivided into Random Access Frames (RAFs) consisting of *K* slots of identical duration. In such a scenario, the input offered load *G* that should be managed by the network can be expressed (in packets per slot) as:

(1) $$G=\chi \;\frac{H}{K},$$

where 0≤χ≤1 is the activation probability, namely, the probability that a user generates a packet in a frame. The transmission in the RAF of each packet is carried out by adopting an SA-based random access scheme combining energy diversity (i.e., NOMA) with packet diversity (i.e., CRDSA, IRSA, …).

Consider first energy diversity. To this aim, identify by *L* the total number of available power levels and define as $E_{l}/N_{0}$ the energy $E_{l}$ used by the *l*-th (l=1,…,L) NOMA level normalized to the noise spectral density $N_{0}$. Besides, assume, for generality, that the *l*-th level may be related to a channel code rate $r_{l}$, thus enabling one to define the vector $r=[r_{1},\ldots ,r_{L}]$ of the code rates associated to all the *L* levels. When a bi-dimensional modulation is chosen to transmit a packet, the corresponding vector of the selected rates (expressed in information bits/symbol) is given by R=2r. When the Shannon bound is assumed, the vector of the Signal-to-Noise Ratio (SNR) thresholds $\alpha =[\alpha_{1},\ldots ,\alpha_{L}]$ required to sustain the rates R can be evaluated by inverting the Shannon bound itself, thus obtaining:

(2) $$\alpha =2^{R}-1,$$

in which the element-wise power function is used to compact the notation. When, instead, a Quadrature Phase-Shift Keying (QPSK) modulation is adopted, the vector of the SNR thresholds can be derived by inverting the expression calculated in [30]. This yields:

(3) $$\alpha =2{\left\{\frac{1}{a_{1}}\left[a_{3}-\log\left(1-\frac{R}{2}\right)\right]\right\}}^{\frac{1}{a_{2}}},$$

where $a_{1}≅1.286$, $a_{2}≅0.931$, and $a_{3}≅0.010$ are proper coefficients. Assume min(α)≥1, so that, for a given energy level, only one successful packet reception is allowed.

Now, let us introduce, in addition to energy diversity, packet diversity. Accordingly, in each RAF, a generic user can send at most one packet that can be repeated *M* times. When just energy diversity is adopted, and hence packet diversity is not considered, we necessarily have M=1. Otherwise, if packet diversity is introduced, M=2 replicas are employed when the CRDSA scheme is used, while, when the IRSA one is adopted, *M* becomes a RV having a Probability Density Function (PDF) that can be optimized through the density evolution method. In summary, when a packet must be transmitted, the source can in general select the *M* value, the relative energy levels, and the *M* slots of the RAF in which the *M* replicas have to be inserted.

**Figure 1:**
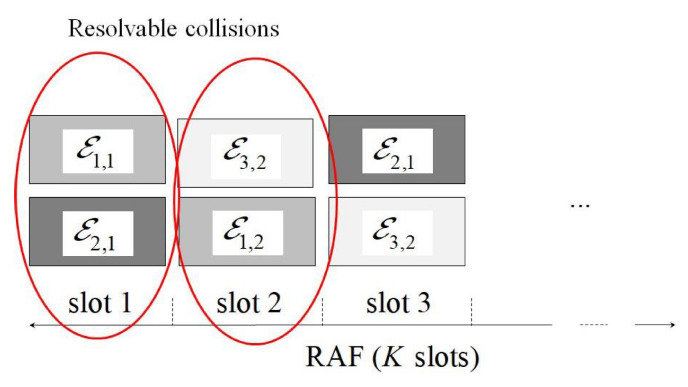
Protocol operation example.

The main protocol operations can be inferred from Figure 1, in which $E_{i,j}$ represents the energy selected by the user *i* for its *j*-th attempt. More precisely, in the reported situation, user 1 chooses the energy level 1 for its first attempt, and the energy level 2 for the second one. We have a collision in the first and in the second slot, since both users choose the same energy level. In the third slot, instead, the transmitters choose different energy levels, thus, according to the NOMA rules, their packets can be successfully decoded by starting from the higher energy one. In this way, the contribution of users 1 and 3 may be (partly) removed from the other slots, hence allowing the correct detection of packet 2 as well by assuming that the remaining interference is within the design threshold. Observe that the adoption of repetition schemes has the advantage, from the user’s viewpoint, of guaranteeing the complete packet delivery up to a certain load, avoiding the need of further retransmissions. This has a positive impact both on system complexity and delay. The joint adoption of repetitions and power diversity allows one to increment the sustainable load. However, the adoption of power diversity may lead to a significant increase of energy consumption, which makes also more critical the impact of imperfect IC on the performance. Therefore, a suitable energy design is required to achieve the joint benefits of repetitions and power diversity, without negative effects on energy consumption and performance.

## 3. Energy Level Design

When energy and packet diversity are jointly employed, the design of the energy levels should be extended with respect to the typical one adopted for the pure NOMA scheme [6]. Moreover, within this design, two further aspects should be carefully considered. The first one concerns the power consumption, since modern communication systems cannot neglect the importance of energy saving. The addressing of such a problem leads to a different perspective in imposing the mathematical constraints on the energy levels, thus moving from the usual requirement on the maximum peak energy [18] to a more suitable one on the average spent energy. For a proper design of the access algorithm, it is hence useful to rely on the average SNR referred to a packet, which can be expressed as:

(4) $$\frac{E_{\mathrm{av}}}{N_{0}}=E\left[M\right]\;\Gamma ,$$

where E[M] denotes the average number of replicas of a packet and:

(5) $$\Gamma =\frac{1}{N_{0}L}\sum_{l=1}^{L}E_{l},$$

represents the average SNR referred to a single replica. The main scope of this paper is to investigate the effects of the available average energy $E_{\mathrm{av}}$ assuming an equally likely energy distribution [8].

The second aspect that should be taken into account in the energy design of a joint NOMA/PR scheme involves the nonidealities of the decoding IC-based process, and in particular the residual interference actually present in a practical receiver due to imperfect cancellation. To ensure the proper algorithm operations in this realistic situation, thus accounting for imperfect IC, limited average energy, and joint power/packet diversity, we propose an energy level design in which the following condition must be met:

(6) $$\frac{\frac{E_{l}}{N_{0}}}{1+\sum_{i=1}^{l-1}\frac{E_{i}}{N_{0}}+\epsilon \left(N_{1}L\Gamma +\sum_{i=l+1}^{L}\frac{E_{i}}{N_{0}}+N_{2}\frac{E_{L}}{N_{0}}\right)}\geq \alpha_{l},\;l=1,\ldots ,L,$$

where 0≤ϵ<1 represents the fraction of the interference remaining after IC (ϵ=1 is not considered since it would imply no IC), $N_{1}\geq 0$ denotes the number of sustainable cancelled transmissions per energy level derived from packet diversity, and $N_{2}\geq 0$ identifies the number of further highest energy sustainable cancelled transmissions. This latter parameter acts as a margin that a designer can introduce to increase the robustness of the decoding process. The condition in (6), which must hold in the slot occupied by a replica to enable its decoding, accounts for the lower (not cancelled) energy levels, and for the residual interference (terms within the round bracket) due to the other (cancelled) replicas deriving from repetition schemes (replicas that are correctly decoded in other slots), and to the higher (cancelled) energy levels in the same slot. By assuming $N_{2}>0$, a large number of further lower energy cancelled transmissions deriving from repetitions may be sustained, if less than $N_{1}+N_{2}$ highest energy cancelled transmissions are present. Note that (6) can be applied also to basic NOMA with imperfect IC, for which $N_{1}=N_{2}=0$, and, furthermore, to basic NOMA with perfect IC, for which ϵ=0. From condition (6), the limiting normalized energies may be determined as:

(7) $$\frac{E_{L}}{N_{0}}=\alpha_{L}\frac{1+L\Gamma \left(1+\epsilon N_{1}\right)}{1+\alpha_{L}\left(1-\epsilon N_{2}\right)},$$

(8) $$\frac{E_{L-1}}{N_{0}}=\left(1+\alpha_{L}\epsilon \right)\frac{\alpha_{L-1}}{1+\alpha_{L-1}}\frac{1+L\Gamma \left(1+\epsilon N_{1}\right)}{1+\alpha_{L}\left(1-\epsilon N_{2}\right)},$$

and so on for the other lower levels. Under the equally likely energy hypothesis, the above equations may be suitably manipulated to evaluate each normalized level, hence obtaining, after some algebra, the normalized energies in a compact form as:

(9) $$\frac{E_{l}}{N_{0}}=\frac{\phi }{\psi }\;\theta_{l},\;l=1,\ldots ,L,$$

where:

(10) $$\phi =1+L\Gamma \left(1+\epsilon N_{1}\right),$$

(11) $$\psi =1+\alpha_{L}\left(1-\epsilon N_{2}\right),$$

(12) $$\theta_{l}=\left(1+\alpha_{L}\right)\delta_{l}\prod_{i=l+1}^{L+1}\beta_{i},\;l=1,\ldots ,L,$$

with:

(13) $$\delta_{l}=\frac{\alpha_{l}}{1+\alpha_{l}},\;l=1,\ldots ,L,$$

(14) $$\beta_{l}=\left\{\begin{array}{cc} \frac{1+\epsilon \alpha_{l}}{1+\alpha_{l}} & l=1,\ldots ,L\\ \;1^{{}^{{}^{{}^{{}^{}}}}} & l=L+1 \end{array}\right.,$$

in which the index L+1 is introduced for mathematical purposes. Note that, being 0≤ϵ<1, we have $0<1/\left(1+\alpha_{l}\right)\leq \beta_{l}<1$ for l=1,…,L. As a further remark regarding this derivation, it is worth observing that (9) represents a general purpose relationship among energies, in which $\theta_{l}$ depends on α and ϵ only, while ϕ and ψ, respectively, depend also on $N_{1}$ and $N_{2}$. Actually, a simplified energy design might be given by:

(15) $$\frac{E_{l}}{N_{0}}=\xi \;\theta_{l},\;l=1,...,L,$$

in which the constant ξ can be determined once the energy probabilities, the transmission scheme, and the average SNR are set. However, the adoption of (9) allows one to relate the energy to the sustainable cancelled patterns, and is, therefore, much more suitable for assessing the performance achievable by a joint NOMA/PR scheme.

In a conventional system, the design parameters are: ϵ (the fraction of interference remaining after IC, dependent on the quality of the receiver), α (the selected rates), $N_{1}$ (the number of cancelled interferers per energy level deriving from repetitions that must be sustained), and $N_{2}$ (the number of further highest energy cancelled interferers deriving from repetitions that must be sustained). Therefore, after the evaluation of ψ by (11), one has to derive Γ, which is necessary to evaluate ϕ in (10). This latter operation can be carried out by substituting (9) and (10) in (4)–(5), and then by solving for Γ, thus obtaining:

(16) $$\Gamma =\frac{\sum_{l=1}^{L}\theta_{l}}{L\left[\psi -\left(1+\epsilon N_{1}\right)\sum_{l=1}^{L}\theta_{l}\right]},$$

which allows the calculation of ϕ by (10) and the subsequent evaluation of the normalized energies by (9). If all levels are associated to the same modulation and code rate, we have $R_{l}=R$ and hence $\alpha_{l}=\alpha$ for l=1,…,L. By consequence, from (14), we obtain:

(17) $$\beta_{l}=\beta =\frac{1+\epsilon \alpha }{1+\alpha },\;l=1,\ldots ,L,$$

which may be usefully inverted as:

(18) $$\alpha =\frac{1-\beta }{\beta -\epsilon }.$$

The adoption of an identical α value simplifies the expressions in (12) and (16), which, by (11) and (18), respectively, become:

(19) $$\theta_{l}=\frac{1-\beta }{\beta -\epsilon }\;\beta^{L-l},\;l=1,\ldots ,L,$$

and:

(20) $$\Gamma =\frac{1-\beta^{L}}{L\left\{1-\epsilon \left[1+N_{2}\left(1-\beta \right)\right]-\left(1+\epsilon N_{1}\right)\left(1-\beta^{L}\right)\right\}}.$$

Concerning this latter formula, one may observe that, since Γ must be positive and β is positive and lower than one, the quantity within the brace must be not negative. This provides the following limiting condition on $N_{1}$:

(21) $$N_{1}\leq \lfloor \frac{\beta^{L}-\epsilon \left[1+N_{2}\left(1-\beta \right)\right]}{\left(1-\beta^{L}\right)\epsilon }\rfloor ,$$

which determines a limit on the maximum IC capability and, in turn, on the throughput.

In some application scenarios characterized by power saving constraints, the average energy, and hence the average SNR, may be considered as a given parameter, in place of the α one adopted in conventional systems based on the rate to support. In these energy-constrained random access networks, (20) may be exploited to evaluate the required α value, given Γ and the other design parameters ϵ, $N_{1}$, and $N_{2}$. To this aim, (20) can be manipulated to derive a polynomial equation in the unknown β as:

(22) $$\beta^{L}+a\beta -b=0,$$

where:

(23a) $$\begin{array}{cc} \; & a=\frac{L\Gamma \epsilon N_{2}}{1+L\Gamma {\left(1+\epsilon N_{1}\right)}_{{}_{{}_{{}_{{}_{}}}}}}, \end{array}$$

(23b) $$\begin{array}{cc} \; & b=\frac{1+\epsilon L\Gamma \left(1+N_{1}+N_{2}\right)}{1+L\Gamma \left(1+\epsilon N_{1}\right)}. \end{array}$$

In general, this equation must be solved numerically, but, in many common situations, such as for L≤4 or $N_{2}=0$, closed-form expressions may be derived. This significantly limits the computational complexity of the design process, making simpler the overall implementation of the derived joint NOMA/PR schemes. Once the β value is obtained (analytically or numerically), one may calculate the corresponding rate α by (18). For example, when $N_{2}=0$, one can easily obtain $\alpha =\left(1-b^{\frac{1}{L}}\right)/\left(b^{\frac{1}{L}}-\epsilon \right)$.

In summary, the main characteristic of the proposed energy design strategy for NOMA/PR schemes consists in enabling the evaluation of the energy levels given the required rates, when power saving is not of concern, or, conversely, the calculation of the rates given the energy levels for power-limited systems.

## 4. Throughput Evaluation

The throughput achievable by a joint power/packet diversity algorithm may be theoretically determined moving from the density evolution method [10]. To this aim, recalling the assumed Poisson arrival process, firstly evaluate the probability of generating *i* packets as a function of the input offered load *G* as:

(24) $$\rho_{i}\left(G\right)=\frac{{\left(E[M]G\right)}^{i-1}}{\left(i-1\right)!}\;\exp\left(-E[M]G\right),\;i=1,2,\ldots$$

where the term E[M]G takes into account that, in the presence of a PR scheme, each generated packet produces, on average, E[M] replicas. Now, to apply density evolution, consider the relationship between the unsolved contentions and the unknown packets (see [10] for the detailed derivation):

(25) $$\lambda \left(q,G\right)=\frac{1}{E[M]}\sum_{M=1}^{\max(M)}M\Lambda \left(M\right){\left[p\left(q,G\right)\right]}^{M-1},$$

in which *q* denotes the probability that a replica is still unknown at the certain stage of the IC process, Λ(M) is the PDF of *M*, and:

(26) p(q,G)=1−∑i=1+∞ρi(G)∑t=0i−1wi,ti−1t1−qtqi−1−t,

represents the probability that a contention in a generic slot is still unresolved. In particular, in (26), $w\left(i,t\right)$ identifies the probability of successfully decoding a replica in a slot with *i* contending ones, *t* of which have been canceled because they have been correctly decoded in other slots. In ideal conditions, that is, with perfect IC (ϵ=0), we have $w\left(i,t\right)=w\left(i-t,0\right)$, whose corresponding approximate expression has been derived in ([27], eq. 18). In such a case, the maximum throughput $G_{\max}$ may be obtained by determining the maximum *G* value for which the inequality q>λ becomes satisfied for 0<q≤1. Observe that, in an asymptotic scenario, that is, for K→∞, all the packets are successfully delivered if the throughput is lower than $G_{\max}$. This condition may be reasonably approached when RAFs composed by a sufficiently large number of slots are selected.

In unideal conditions, that is, with imperfect IC (ϵ>0), $w\left(i,t\right)$ may be determined as a function of $N_{1}$ and $N_{2}$ for a given set of parameters. For example, $w\left(i,i-1\right)=1$ for $i<N_{1}+N_{2}+1$, being this limit the guaranteed IC capability. In a more general condition, we have $w\left(i,t\right)<w\left(i-t,0\right)$, being the main performance reduction due to the replicas associated to the lower energy levels, which cannot sustain the partially cancelled energy of the replicas associated to the higher energy levels. For the developed energy design, the $w\left(i,t\right)$ values may be exhaustively evaluated. However, with partial IC, the density evolution approach cannot be directly used as in [10] to assess the limiting performance. In fact, the full packet delivery is not feasible, given that the density evolution analysis relays on the hypothesis $w\left(i,i-1\right)=1$, which cannot be achieved for *i* values exceeding a threshold dependent on the parameter set. Hence, the condition q>λ cannot be guaranteed for very low *q* values. Actually, the achievable throughput depends on energy, and, as a consequence, on $N_{1}$ and $N_{2}$ for a given scenario (access algorithm, ϵ value, number of energy levels). A general treatise would require a modification of the density evolution approach. This task is out of the scope of this paper, which focuses on the energy requirements that allow the access algorithm to approach its limiting performance, thus ensuring the almost complete delivery of the packets within the maximum throughput for sufficiently large *K* values. To deal with this specific problem one may exploit the fact that, by progressively increasing the $N_{1}$ and $N_{2}$ values, the limiting throughput $G_{\max}$ may be approached when the condition q>λ is guaranteed for $q_{0}<q\leq 1$ with a sufficiently low $q_{0}$ value, which in this study is selected equal to 0.05. Note that this latter achievement is guaranteed for $\epsilon^{\prime }<\epsilon$, that is, for all residual fractions of interference $\epsilon^{\prime }$ lower than the design value ϵ. This implies that, if the receiver has IC capabilities better than those assumed during the design, it can sustain a certain fluctuation of the energy level, deriving, for example, from fading.

The conceived throughput estimation is applied to the NOMA/CRDSA and NOMA/IRSA algorithms, which are of main interest because of the implementation of the CRDSA and IRSA schemes in last generation satellite systems [14]. In particular, for the NOMA/CRDSA algorithm, we simply have Λ(2)=1 and Λ(M)=0 for M≠2 [9], since *M* is deterministic. In the NOMA/IRSA case, instead, *M* is a RV, whose PDF have been determined in [18] assuming max(M)=8. For this protocol, named NOMA/IRSA-8 from now on, the energy requirements are very severe and the limiting performance may be approached just using a very large number of slots. Hence, in this paper, we also adopt a simpler version characterized by max(M)=3, named NOMA/IRSA-3, which allows a more quick identification of the energy required to approach $G_{\max}$. Table 1 summarizes the parameters adopted for these two schemes.

## 5. Results

As a first set of results, Table 2 reports the achievable limiting throughput and the required average SNR in the presence of perfect IC (ϵ=0) for the three algorithms under investigation (NOMA/CRDSA, NOMA/IRSA-3, NOMA/IRSA-8). The values are obtained assuming R=1 information bits per symbol, using the Shannon model (for which also α=1), and selecting the parameters in Table 1. In particular, Table 2 shows that, for a given scheme, the increase of the number of levels provides, as expected, a higher throughput, but at the cost of an increased average energy (higher power consumption). Throughput improvements may be also obtained, for a given *L* value, by increasing the level of packet diversity, still spending a higher energy.

**Figure 2:**
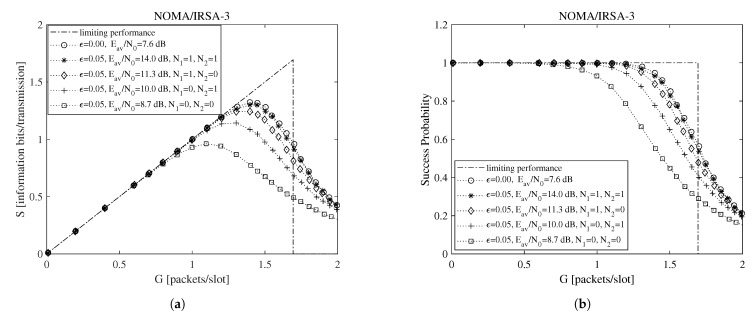
Performance as a function of the channel load for NOMA/IRSA-3 with K=100, L=3, and R=1 information bits per symbol: (**a**) throughput, (**b**) success probability.

**Figure 3:**
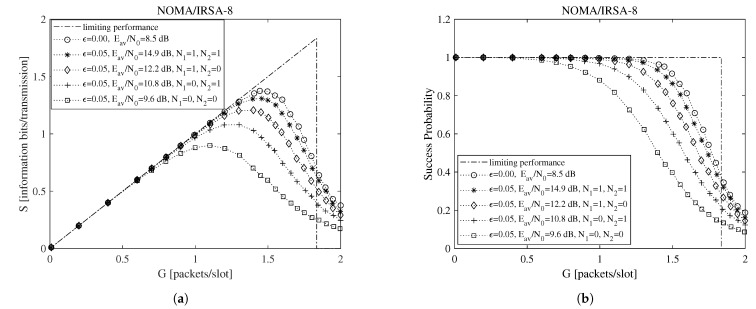
Performance as a function of the channel load for NOMA/IRSA-8 with K=100, L=3, and R=1 information bits per symbol: (**a**) throughput, (**b**) success probability.

To begin the exploration of the performance under imperfect IC when the Shannon bound is adopted, consider Figure 2 and Figure 3, which report, for ϵ=0.05, L=3, K=100, and R=1 information bits per symbol, the throughput for the NOMA/IRSA-3 (Figure 2a) and the NOMA/IRSA-8 (Figure 3a) schemes, together with the corresponding success probabilities (Figure 2b and Figure 3b).

**Figure 4:**
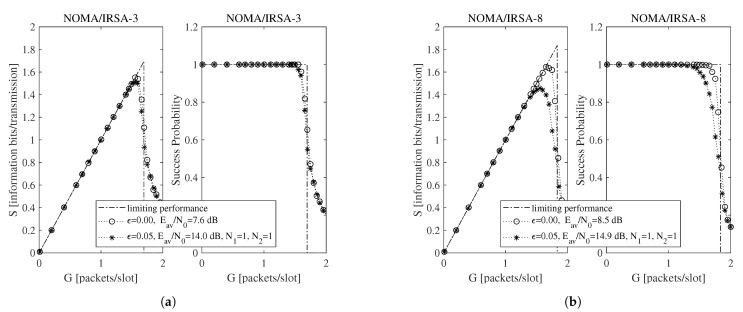
Throughput and success probability as a function of the channel load for K=1000, L=3, and R=1 information bits per symbol: (**a**) NOMA/IRSA-3, (**b**) NOMA/IRSA-8.

**Figure 5:**
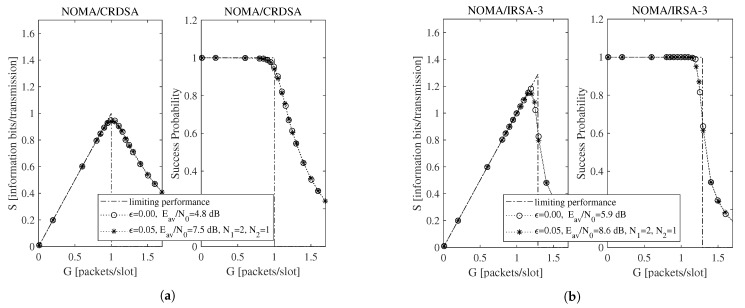
Throughput and success probability as a function of the channel load for K=1000, L=2, and R=1 information bits per symbol: (**a**) NOMA/CRDSA, (**b**) NOMA/IRSA-3.

Beside the analytical plot representing the limiting performance (dash-dotted line) and the perfect IC case (dotted line with circle marker), each figure shows a set of simulated curves derived by selecting different $N_{1}$ and $N_{2}$ values. Both analysis and simulations are implemented in Matlab. In particular, the simulations are carried out by generating the Poisson traffic for a given load *G*, assigning to each packet the proper *M* and energy values, and then randomly locating the replicas in the RAF. Each simulated point is calculated by averaging the results obtained from $N_{\mathrm{sim}}$ = 10,000 RAF realizations. From these figures one may observe that, for NOMA/IRSA-3, the limiting performance is approached using $N_{1}=1$ and $N_{2}=1$, while, for NOMA/IRSA-8, higher values would be required. Besides, for a given $\left(N_{1},N_{2}\right)$ pair, the performance of NOMA/IRSA-8 is comparable to that of NOMA/IRSA-3, despite the higher energy level and the higher limiting performance of the first scheme. Similar arguments hold also for K=1000, that is, when longer RAFs are considered, as it may be noticed from Figure 4 and Figure 5.

**Figure 6:**
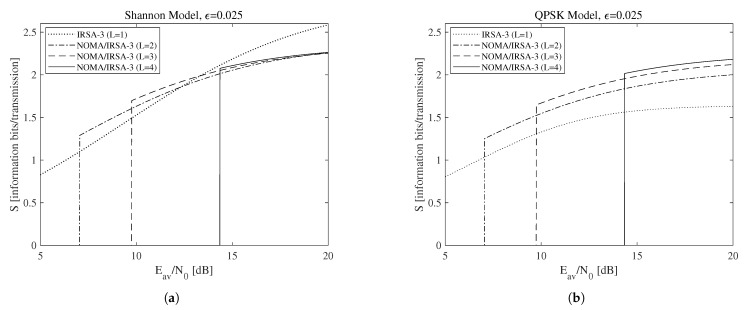
Throughput as a function of the available average SNR for ϵ=0.025: (**a**) Shannon bound, (**b**) QPSK modulation.

According to the above discussion, let us now focus on the relationship between the sustainable rate and the available energy. More precisely, consider the minimum threshold α=1, achievable by selecting the minimum average SNR ${\left(E_{\mathrm{av}}/N_{0}\right)}_{\min}$, and the maximum sustainable threshold $\alpha_{\max}$, obtainable from (22) and (18) when $E_{\mathrm{av}}/N_{0}\to \infty$ and hence Γ→∞. These parameters, together with the maximum sustainable rate $R_{\max}$, derived by (2) for the Shannon bound and by (3) for the QPSK modulation, are reported in Table 3 for each of the analyzed scenarios, that is, when the access algorithm and the parameters *L*, ϵ, $N_{1}$, and $N_{2}$ are identified. In this table, the $\left(N_{1},N_{2}\right)$ pairs are properly selected according to the method explained in the previous section, which allows the analyzed algorithms to approach $G_{\max}$ for a sufficiently large *K* value. It is worth to observe, firstly, that ${\left(E_{\mathrm{av}}/N_{0}\right)}_{\min}$ and $\alpha_{\max}$ do not depend from the chosen modulation bound, and, secondly, that all the values in the table have been calculated in closed-form, since all the involved scenarios are characterized by L≤4 and/or $N_{2}=0$, thus enabling to analytically solve (22). Among the values in the table, let us start by considering those corresponding to ${\left(E_{\mathrm{av}}/N_{0}\right)}_{\min}$ for NOMA/IRSA-3 and ϵ=0.025 without constraints on the code rate and on the modulation complexity, hence allowing the usage of all the rates complying with α>1. The throughput achievable in these scenarios for different values of *L* is reported in Figure 6a for the Shannon bound and in Figure 6b for the QPSK modulation. More precisely, Figure 6a interestingly reveals that the simple IRSA-3 algorithm (L=1), in which NOMA is not adopted, may anyway achieve a satisfactory performance, close or, in some cases, even better than that provided by the combined schemes. This is due to its low energy requirements and to the possibility of using complex modulations, which are implicitly available when the Shannon bound is assumed. A realistic setting involving the QPSK modulation (R≤2) modifies the previous conclusion, putting into evidence that the increase of the number of energy levels enables the NOMA/IRSA-3 scheme to better exploit its higher sustainable load.

**Figure 7:**
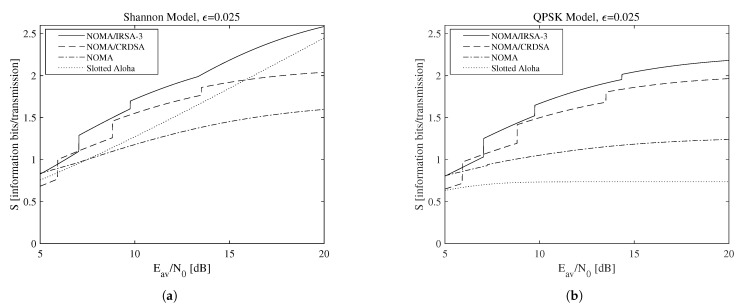
Maximum throughput as a function of the available average SNR for ϵ=0.025: (**a**) Shannon bound, (**b**) QPSK modulation.

A more general set of results is reported in Figure 7, which shows the achievable throughput, that is, the maximum throughput calculated over all the available *L* values for NOMA/IRSA-3, NOMA/CRDSA, pure NOMA, and SA when the Shannon bound (Figure 7a) and the QPSK modulation (Figure 7b) are adopted. The performance of the pure NOMA algorithm (M=1 and L>1) is shown separately from that of the SA one (M=L=1), since the latter does not use IC and, by consequence, does not depend on ϵ. When the Shannon bound is adopted (i.e., R>1), thus no limitations on the code rate and on the modulation complexity are present, the simple SA, despite its low sustainable rate, is able to achieve an interesting performance by exploiting its very low energy requirements. When QPSK modulation is instead assumed, the repetition schemes perform significantly better and are definitely preferable. However, despite the specific modulation model, the algorithms adopting repetition schemes, that is, NOMA/IRSA and NOMA/CRDSA (even in their base version with L=1), allow the transmitters to deliver all their packets in a sufficiently long RAF, while pure NOMA (including SA) necessarily requires retransmissions in successive RAFs to recover the failed attempts.

**Figure 8:**
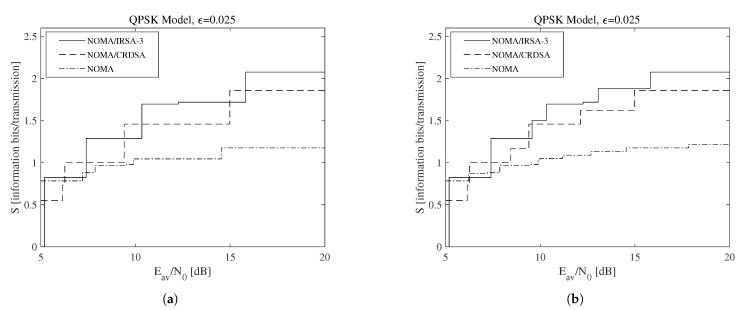
Throughput as a function of the available average SNR for ϵ=0.025 and a discrete set of code rates adopting the QPSK modulation: (**a**) single code rate, (**b**) double code rate.

**Figure 9:**
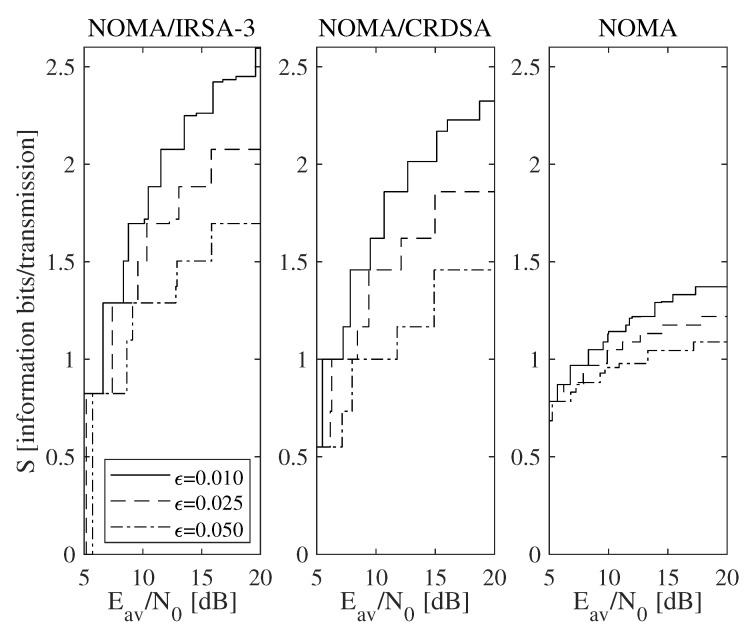
Throughput as a function of the available average SNR for different ϵ values assuming the QPSK modulation and a discrete set of code rates.

To make the presented discussion more adherent to a practical context, consider now that the code rate $r_{l}$ for the *l*-th energy level can be selected in a finite set R∈{1/2,2/3,3/4,5/6}, whose corresponding average SNRs referred to a single replica may be determined by (16). The resulting achievable throughput is reported in Figure 8, which is obtained for ϵ=0.025 when the QPSK modulation is used. In particular, Figure 8a refers to the scenario in which a single code rate is adopted by all the energy levels (i.e., $r_{l}=r\in R$ for l=1,…,L), while Figure 8b refers to the scenario in which a double code rate is allowed, since a higher code rate may be used by the lowest energy level for compensation (i.e., $r_{l}=r\in R$ for l=2,…,L and $r\leq r_{1}\in R$). Both subfigures reveal that, in a discrete-rate situation, the algorithms that offer the best performance are influenced by the available energy. Besides, observe that all the results in Figure 8 are bounded by those in Figure 7b. A more specific view, referred to the NOMA/IRSA-3 scheme, is available in Table 4, which lists a suitable combination of rates and the corresponding limiting performance. Finally, a complete evaluation, considering the multi-rate design for different ϵ values is shown in Figure 9. From this figure, we may infer that just the multi-rate design allows one to approach the continuous throughput curve, and that the distinction about the performance of the different algorithms becomes clearer. In particular, the throughput improvement deriving from the repetition schemes strongly depends on the residual amount of interference remaining after the IC process.

## 6. Conclusions

The joint adoption of NOMA and packet diversity in the presence of imperfect IC and constraints on the average available energy has been investigated. A suitable energy design allows one to achieve the benefits of repetitions and power diversity, without negative effects on energy consumption, which may affect the user’s experience. The relationship between the energy levels and the rates sustainable by the system has been theoretically derived to enable the design of both rate-based and energy-constrained random access networks. Besides, a throughput analysis moving from the density evolution approach has been developed to identify the limiting performance of NOMA/CRDSA and NOMA/IRSA schemes.

The obtained results have shown that the NOMA/PR strategy allows significant throughput improvements with respect to the pure NOMA and PR algorithms, but have confirmed also that the residual interference may severely affect the performance of solutions previously conceived assuming perfect IC. A comparison carried out considering an ideal Shannon model and a more realistic QPSK modulation in a practical scenario characterized by discretized code rates has revealed that, in the first case, the basic SA scheme is competitive with the more sophisticated NOMA/PR ones, while, in the second case, the joint usage of energy and packet diversity is highly preferable. To exploit this capability, some possible code rate combinations have been explored by taking into account different levels of cancelled interference. This has more clearly put into evidence the applicability of the developed energy design, which may hence represent a useful tool for introducing energy saving in next-generation satellite and terrestrial systems, while maintaining a satisfactory performance for the corresponding random access channels.

Current research efforts are devoted to deepen the behavior of the NOMA/PR schemes in the low SNR regime, in which the correct detection of more than one packet in a single attempt is possibile. The modifications of the energy design in these situations is one of the activities carried out in the present research activity.

## Figures and Tables

**Table 1:** PDF of the number *M* of replicas as a function of the number *L* of energy levels for the considered NOMA/IRSA schemes.

*L*	NOMA/IRSA-3		NOMA/IRSA-8			
	M		M			
	**2**	**3**	**2**	**3**	**4**	**8**
1	0.179	0.821	0.5112	0.2660		0.2228
2	0.408	0.592	0.6607	0.1605		0.1788
3	0.519	0.481	0.7439	0.0906	0.0156	0.1499
4	0.581	0.419	0.7947	0.0470		0.1583
5	0.618	0.382	0.8370			0.1630

**Table 2:** Limiting throughput $G_{\max}$ and required SNR $E_{\mathrm{av}}/N_{0}$ as a function of the number *L* of energy levels in ideal conditions for R=1 information bits/symbol and assuming the Shannon bound.

*L*	CRDSA		NOMA/IRSA-3		NOMA/IRSA-8	
	$G_{\max}$	$\;E_{\mathrm{av}}/N_{0}$ [dB]	$G_{\max}$	$\;E_{\mathrm{av}}/N_{0}$ [dB]	$G_{\max}$	$\;E_{\mathrm{av}}/N_{0}$ [dB]
1	0.55	3.01	0.82	4.50	0.94	5.57
2	1.00	4.77	1.29	5.90	1.43	6.86
3	1.46	6.69	1.69	7.63	1.86	8.48
4	1.86	8.75	2.07	9.58	2.27	10.51
5	2.23	10.93	2.43	11.69	2.66	12.66

**Table 3:** Relationship between energy requirement and sustainable rate for different scenarios.

*L*	ϵ	$N_{1}$	$N_{2}$	${\left(E_{\mathrm{av}}/N_{0}\right)}_{\min}$ [dB]		$\alpha_{\max}$	$R_{\max}$ [Information Bits/Symbol]	
				NOMA/CRDSA	NOMA/IRSA-3		Shannon	QPSK
1	0.05	4	0	3.98	5.47	5.00	2.58	1.90
2		2	1	7.48	8.60	1.68	1.42	1.32
3		1	1	13.06	14.00	1.15	1.10	1.06
1	0.025	4	0	3.47	4.96	10.00	3.46	1.99
2		2	1	5.92	7.05	2.59	1.84	1.61
3		1	1	8.82	9.75	1.61	1.38	1.29
4		1	0	13.52	14.34	1.19	1.13	1.09
1	0.01	4	0	3.19	4.68	25.00	4.70	2.00
2		2	1	5.20	6.32	4.40	2.43	1.86
3		1	1	7.42	8.36	2.43	1.78	1.57
4		1	0	10.10	10.93	1.71	1.44	1.34
5		1	0	14.68	15.44	1.22	1.15	1.10

**Table 4:** Discrete code rate/energy level designs for the NOMA/IRSA-3 scheme with ϵ=0.025.

*L*	$r_{1}$	$r_{2}$	$r_{3}$	$r_{4}$	*R* [Information Bits/Symbol]	$G_{\max}$	*S*	$E_{\mathrm{av}}/N_{0}$ [dB]
1	1/2				1.00	0.82	0.82	5.18
2	1/2	1/2			1.00	1.29	1.29	7.39
2	2/3	1/2			1.17	1.29	1.50	9.57
3	1/2	1/2	1/2		1.00	1.69	1.69	10.34
2	2/3	2/3			1.33	1.29	1.72	12.26
3	2/3	1/2	1/2		1.11	1.69	1.88	13.06
4	1/2	1/2	1/2	1/2	1.00	2.07	2.07	15.81

## Abbreviations

The following abbreviations are used in this manuscript:

CRDSA	Contention Resolution Diversity SA
IC	Interference Cancellation
IoT	Internet of Things
IRSA	Irregular Repetition SA
NOMA	Non-Orthogonal Multiple Access
QPSK	Quadrature Phase-Shift Keying
PDF	Probability Density Function
PR	Packet Repetition
RAF	Random Access Frame
RV	Random Variable
SA	Slotted Aloha
SNR	Signal to Noise Ratio
